# Participation of Children With Developmental Language Disorders in Educational Settings—Parents' Perspectives on Patterns, Environmental Influences and Support Strategies

**DOI:** 10.1111/cch.70310

**Published:** 2026-07-03

**Authors:** Katharina Kuhlmann, Ulla Licandro

**Affiliations:** ^1^ School of Educational and Social Sciences, Department of Special Needs Education and Rehabilitation Carl von Ossietzky Universität Oldenburg Oldenburg Germany

**Keywords:** (pre)school participation, developmental language disorder, environment, involvement, parents, PEM‐CY, questionnaire

## Abstract

**Background:**

Participation in educational settings is essential for children's development, learning and well‐being. Although access to (pre)school is a fundamental right, participation often depends on children's language abilities. Developmental language disorder (DLD) is among the most common childhood conditions, yet little is known about age‐ and institution‐related differences in participation among children with DLD. This study explores parental perspectives on participation of children with DLD in preschool and primary school settings, considering environmental influences and strategies used to support participation.

**Methods:**

Parents of preschool‐aged (*n* = 45; *M*_*age*_ = 6.3) and primary school‐aged children (*n* = 56; *M*_*age*_ = 8.5) with DLD in Germany completed the Participation and Environment Measure for Children and Youth. Mann–Whitney *U* tests and chi‐squared analyses were used to examine group differences, whereas an inductive content analysis was applied to identify categories in the responses to open‐ended questions.

**Results:**

According to parental reports, primary school children showed slightly higher average participation, but only two activities differed significantly, indicating largely similar participation patterns across groups. Despite high overall involvement, participation frequency varied, with almost a third of children never taking part in school‐sponsored teams or holding special roles. Classroom activities showed the highest participation frequency but also the lowest involvement and the strongest parental desire for change. Environmental supports outweighed barriers, but sensory and cognitive demands remained key challenges across both groups. Strategies of preschool parents focused on daily routines, planning and logistics, whereas parents of school‐aged children emphasized academic support and collaboration with educators. Across both groups, emotional and motivational support was the most reported strategy.

**Conclusion:**

Parental reports highlight that participation among children with DLD is shaped more by environmental demands and available supports than by educational stage alone. These findings underscore the importance of context‐sensitive, family‐informed approaches to fostering meaningful engagement in everyday school activities.

## Summary

- Participation patterns among preschool and school‐aged children varied widely across activities—from nonparticipation to highest frequency, paired with lowest involvement and strongest desire for change—highlighting that participation extends beyond physical presence.
- Reported participation patterns for both groups—children with DLD in preschool and in primary school—were similar, with over a quarter of parents expressing a desire for change, primarily wishing for higher frequency and greater involvement.
- Environmental supports were perceived to be more than three times as frequent as barriers, yet sensory and cognitive challenges emphasize the need for individualized adjustments in educational settings.
- Parents used a range of strategies to support their children's participation in educational settings, addressing both environmental/contextual and intrinsic factors.
- Parental perspectives on (pre)school participation provide valuable insights into children's needs.

## 1 Introduction

Participation plays a central role in children's development, health and overall well‐being (WHO 2007). As a multidimensional construct, it encompasses not only physical presence (*attendance*) but also the subjective experience of participation while attending (*involvement*), including elements such as motivation, social connection and affect (Imms et al. 2016). The evidence‐based family of participation‐related constructs (fPRC) framework further conceptualizes participation as shaped by dynamic interactions between intrinsic child factors (i.e., activity competence, sense of self and preferences) and extrinsic factors (i.e., environment and context) (Imms et al. 2017).

Given the substantial amount of time children spend in school, the school environment is especially important for enabling and maintaining participation (John‐Akinola and Nic‐Gabhainn 2014). With the ratification of the United Nations Convention on the Rights of Persons with Disabilities (UNCRPD), Germany committed to developing an inclusive education system that ensures equal participation in all school‐related activities, regardless of children's individual competencies or differences (United Nations 2006). Even prior to school entry, it is fundamental to protect and encourage children's involvement during preschool years (Correia et al. 2019; United Nations Committee on the Rights of the Child 2005). Although the right to (pre)school participation is unconditional, its realization often depends on children's language and communication skills (Chow et al. 2022; Gibbons et al. 2022). In the classroom, language serves multiple roles: It is a subject of learning, a medium through which learning takes place and a carrier of knowledge that allows information to be communicated (e.g., Snow and Uccelli 2009). Beyond classroom instruction, extracurricular activities such as socializing with peers, taking on special roles (e.g., classroom helper), participating in events (e.g., performances) and engaging in organizations (e.g., working groups) also heavily rely on language skills.

The relationship between participation and language raises questions about the participation of children with developmental language disorder (DLD) in educational activities. Approximately 7.6% of children have DLD (Norbury et al. 2016), a condition characterized by difficulties in language comprehension and/or production affecting one or more linguistic domains, without a known biomedical cause (Bishop et al. 2017). DLD is frequently linked to a range of challenges, including lower academic achievement (Ziegenfusz et al. 2022), difficulties in peer interactions (Lloyd‐Esenkaya et al. 2020), a higher risk of peer victimization (van den Bedem et al. 2018) and increased behavioral problems (Yew and O'Kearney 2013). In a Swedish interview study, adolescents with DLD aged 13–19 years (*n* = 23) reported negative emotions in school‐based communication situations (Ekström et al. 2023). For example, the fear of saying something wrong led participants to avoid speaking up in class, and comparisons with peers reinforced their feeling of reduced competence. However, not all children with DLD display specific difficulties beyond the reception and production of language, which highlights the heterogeneity within the group. Despite the high prevalence of DLD, there has been limited systematic investigation on participation patterns and environmental factors that support or hinder children's participation across (pre)school‐related activities. Quantitative studies on (pre)school participation often combine different types of impairments into a single sample and compare the results to children without impairments (Jeong 2020; Coster et al. 2013). Although this approach highlights group differences, it also restricts the ability to draw nuanced conclusions for specific populations.

Previous research involving parents of children with disabilities demonstrates that parents are well positioned to assess their children's (pre)school participation (Benjamin et al. 2017; Coster et al. 2013). Although parents are not present in the classroom, their perspectives are informed by multiple sources of indirect information (e.g., communication with teachers, children's reports and parents' long‐term knowledge of their child across educational contexts), which contribute to a holistic understanding of their children's needs, beyond classroom‐based observations. However, the perspectives of parents of children with DLD remain underrepresented in (pre)school participation research.

To date, only one study has specifically examined parental perceptions of school participation in a small sample of children with DLD in Germany: Opitz (2024) found no significant differences in the frequency of school participation between 7‐ to 10‐year‐old children with DLD (*n* = 17) and their peers without DLD (*n* = 25). Although involvement was rated slightly higher for children with DLD, this trend was not statistically significant. Complementary evidence from an Australian parent‐report study by Farrell et al. (2025) suggests a similar pattern for organized extracurricular social activities (OESAs), with no significant group differences in participation breadth, intensity or total number between 4‐ to 12‐year‐old children with DLD (*n* = 18) and their typically developing peers (*n* = 21).

Given that these studies have reported only limited differences in participation between children with DLD and typically developing peers (Opitz 2024; Farrell et al. 2025), it remains important to examine whether variability in participation exists within the heterogeneous group of children with DLD, which may be obscured in overall group‐level comparisons.

As previous research has reported age‐related differences in school participation in favour of older children (Krieger et al. 2023; Simpson et al. 2018), this may suggest that such differences are influenced by changing institutional demands, highlighting the relevance of examining participation at different educational stages. Given that participation does not begin at school age but is already highly relevant during the preschool years (Correia et al. 2019), the present study focuses on a comparison of participation between preschool and primary school contexts among children with DLD.

Although preschool and school represent distinct educational contexts with different structural and pedagogical characteristics within the German education system, both constitute central settings of early educational participation. They share key participation demands, such as engagement in group routines, peer interactions, communication with adults and involvement in structured learning activities. Examining participation across preschool and school allows for a developmental and contextual perspective on how participation may differ between educational stages in the context of the transition to formal schooling. This transition is particularly relevant for children with DLD, as linguistic, social and academic demands typically increase in school settings. A comparative approach therefore enables the identification of context‐specific and cross‐contextual aspects of participation.

Against this background, the present study aims to provide a nuanced understanding of the participation experiences of children with DLD at the end of preschool and in primary school, based on parent reports. This research was guided by the following questions:

1. What participation patterns do parents report for preschool and primary school children with DLD in educational settings?
2. What environmental factors do parents identify as supports and barriers to participation in these settings?
3. What strategies do parents report to support their children's participation in these educational settings?

## 2 Methods

### 2.1 Research Design

A cross‐sectional study design was employed, incorporating both quantitative and qualitative methods. Quantitative, descriptive survey data were collected to address the first two questions. To explore the third research question, qualitative data were obtained through responses to an open‐ended survey item. Ethical approval for the study was obtained from the university's institutional ethics committee. Written informed consent was obtained prior to questionnaire administration and child assessment.

### 2.2 Participants

Participants were recruited between March and September 2023 at an inpatient rehabilitation clinic providing a structured 4‐week therapy programme for children with speech, language and communication needs from across Germany.

In the present study, parents of preschool and school‐aged children (5–10 years) with a language‐related ICD‐10‐GM (German Modification) diagnosis (F80.0, F80.1, F80.2) were invited to participate, as ICD‐11 has not yet been implemented in routine clinical practice in Germany. The diagnosis had to be established by a paediatrician prior to referral to the rehabilitation clinic. In rare instances (*n* = 10) where residual categories (F80.8 and F80.9) had been assigned, eligibility was determined through a review of existing clinical records to ensure that persisting language difficulties constituted the primary condition. For the purposes of the present study, ICD‐10‐GM diagnostic subcategories were not analysed separately, as the research focus was on participation patterns within the broader DLD construct rather than on differences between specific diagnostic codes.

Although ICD‐10‐GM does not use the term DLD, the clinical characteristics reflected in its F80 categories—namely, persistent language difficulties that are not attributable to other known conditions and may be associated with academic and social challenges—broadly correspond to the construct of DLD as defined in the ICD‐11 diagnostic framework and in the CATALISE consensus (Bishop et al. 2017). We therefore use the term DLD throughout the manuscript to reflect the current international terminology.

Beyond existing clinical diagnosis, children were required to score below the mean (−1.25 SD) on at least one standardized language test or to exhibit phonological processes that deviated from age‐appropriate norms. Additionally, children were required to demonstrate average cognitive abilities, as assessed by a short‐form nonverbal test, within the 95% confidence interval, in light of the ongoing lack of consensus in German‐speaking countries concerning the role of mild cognitive impairments in DLD (Lüke et al. 2023).

The final sample comprised 101 participants: 45 parents of children with DLD at the end of preschool (*M* = 6.3, SD = 0.3) and 56 parents of school‐aged children with DLD (*M* = 8.5, SD = 1.2). There were no significant differences in the demographic profiles of preschool and school‐aged children (see Table 1). Table 2 summarizes the results of standardized tests, showing no significant differences between preschool and school‐aged children in terms of nonverbal cognitive abilities, phonological processing, receptive grammar or receptive vocabulary. Differences in phonological processes and expressive vocabulary were expected, given the children's age and the specific assessment tools used. Phonological processes are more common in younger children and typically decrease with maturation, whereas vocabulary measures in older age groups may demonstrate reduced discrimination in the lower performance range. As differences in specific language profiles do not necessarily translate directly into differences in participation, comparisons between preschool and school‐aged children were conducted to examine participation as a functional outcome.

**TABLE 1:** Characteristics of participating parents and their children.

Demographic variables	Total (*n* = 101)	Preschool (*n* = 45)	School (*n* = 56)	*p*
	% (*n*)	% (*n*)	% (*n*)	
Child's gender				0.394
Male	64.4 (65)	68.9 (31)	60.7 (34)	
Female	35.6 (36)	31.1 (14)	39.3 (22)	
Bilingualism^a^				0.561
Yes	21.8 (22)	24.4 (11)	19.6 (11)	
No	78.2 (79)	75.6 (34)	80.4 (45)	
Responding parent				0.901
Mother	87.1 (88)	86.7 (39)	87.5 (49)	
Father	12.9 (13)	13.3 (6)	12.5 (7)	
Federal states of Germany				0.166
Lower Saxony	31.7 (32)	42.9 (18)	25.0 (14)	
North Rhine‐Westphalia	24.8 (25)	19.0 (8)	30.4 (17)	
Hesse	8.9 (9)	11.9 (5)	7.1 (4)	
Other	31.7 (32)	26.2 (11)	37.5 (21)	
Missing	3.0 (3)	6.7 (3)	0.0 (0)	
Mothers highest level of education				0.543
No degree or elementary education	1.0 (1)	0.0 (0)	1.8 (1)	
Lower secondary certificate (9 years)	11.9 (12)	11.1 (5)	12.5 (7)	
Intermediate school certificate (10 years)	48.5 (49)	46.7 (21)	50.0 (28)	
Higher education entrance qualification	20.8 (21)	17.8 (8)	23.2 (13)	
College or university degree	17.8 (18)	24.4 (11)	12.5 (7)	
Fathers highest level of education				0.764
No degree or elementary education	2.0 (2)	2.3 (1)	1.8 (1)	
Lower secondary certificate (9 years)	20.8 (21)	15.9 (7)	25.5 (14)	
Intermediate school certificate (10 years)	36.6 (37)	36.4 (16)	38.2 (21)	
Higher education entrance qualification	21.8 (22)	25.0 (11)	20.0 (11)	
College or university degree	16.8 (17)	20.5 (9)	14.5 (8)	
Missing	2.0 (2)	2.2 (1)	1.8 (1)	
MacArthur scale (Adler et al. 2000)				0.586
Subjective social status (1–10)^b^	6.18 (1.3)	6.27 (1.3)	6.11 (1.3)	
Missing	6.9 (7)	8.9 (4)	5.4 (3)	

*Note:* *p* values are based on Fisher's exact tests, chi‐square tests or Mann–Whitney *U* tests, depending on the scale level and distribution of the variable.

^a^ Children were simultaneous bilinguals and received systematic educational exposure for at least 2.5 years.

^b^ Response options ranged from 1 to 10, with higher values indicating higher subjective social status; values are reported as *M* (SD).

**TABLE 2:** Descriptive results of administered test instruments.

Measure	Preschool			School			*p*
	*n*	*M* (SD)	Range	*n*	*M* (SD)	Range	
Cognitive ability (IQ)	45	100.0 (12.1)	81–131	56	97.6 (12.4)	74–133	0.351
Nonword repetition (*T* score)	41	32.0 (8.8)	17.0–55.0	53	32.8 (10.9)	17.0–64.8	0.586
Expressive vocabulary (*T* score)	41	42.5 (11.1)	0–62	55	29.1 (19.5)	0–58	0.001
Receptive grammar (*T* score)	45	45.0 (11.0)	25–67	56	43.0 (11.1)	23–64	0.365

	*n*	Mdn (Q1–Q3)	Range	*n*	Mdn (Q1–Q3)	Range	
Receptive vocabulary (percentile rank)	45	22 (3–46.5)	0–100	56	22 (4–48)	0–100	0.742

	*n*	% (*n*)		*n*	% (*n*)		
Phonological processes % (≥ 1 atypical process)	45	91.1 (41)		55	52.7 (29)		0.001

*Note:* Cognitive ability was assessed using the Wechsler Nonverbal Scale of Ability (WNV; Petermann 2014); nonword repetition using the Mottier test (Mottier 1951; Wild and Fleck 2013); expressive and receptive vocabulary using the Word Range and Word Retrieval Test for 6‐ to 10‐Year‐Olds (WWT 6–10; Glück 2011); receptive grammar using the Test for the Reception of Grammar (TROG‐D; Fox‐Boyer 2020); and phonological processes using the Psycholinguistic Analysis of Childhood Speech Sound Disorders (PLAKSS‐II; Fox‐Boyer 2014). Sample sizes vary across measures due to missing data. *p* values are based on chi‐square tests, Mann–Whitney *U* tests or independent *t* tests, depending on the scale level and distribution of the variable.

### 2.3 Participation Measurement

The cross‐culturally adapted German paper‐pencil version of the Participation and Environment Measure for Children and Youth (PEM‐CY[G]) was used for data collection (Krieger et al. 2020; Coster et al. 2011). The PEM‐CY is a parent‐report questionnaire designed to assess participation patterns and environmental influences on children aged 5–17 years across three settings: home, school and community. In alignment with the research focus of the current study, only the (pre)school setting was analysed. To accommodate the inclusion of 5‐ and 6‐year‐old children, the word ‘kindergarten’ was added to the school setting, as in the German adaptation (Krieger et al. 2020). The participation section includes five items, representing typical (pre)school activities. For each activity, parents rated their child's participation along three dimensions: (1) *frequency* (8‐point scale, from *never* [0] to *daily* [7]); (2) *level of involvement* (5‐point scale, from *minimally involved* [1] to *very involved* [5]; this item was omitted if parents indicated the activity was never performed); (3) *desire for change* (*no*/*yes*, with five follow‐up questions if “yes”).

Environment influences were assessed using nine items that asked parents whether specific environmental features supported or hindered their child's participation in (pre)school activities. Response options included *not an issue*, *usually helps*, *sometimes helps/sometimes makes harder* and *usually makes harder*.

To explore parental support strategies, the PEM‐CY also includes an open‐ended question inviting parents to list up to three strategies they use to support their child's participation in (pre)school settings. The PEM‐CY is grounded in the International Classification of Function, Disability and Health (ICF) framework (Chien et al. 2014) and demonstrates moderate to good internal consistency and test–retest reliability (Coster et al. 2011).

### 2.4 Data Analysis

To address Research Questions 1 and 2, quantitative analyses were conducted using IBM SPSS Statistics (Version 29.0). Children were grouped by educational stage (preschool and school‐aged), and parental perceptions on participation and environmental items were compared. The Kolmogorov–Smirnov test indicated nonnormal distribution of the PEM‐CY data. Accordingly, Mann–Whitney *U* tests were used for ordinal variables (i.e., *participation frequency* and *involvement*), whereas chi‐squared analyses were applied to categorical variables (i.e., *never participates*, *desire for change* and *environmental supports/barriers*). When more than 20% of expected cell frequencies were below 5, or any were below 1, Fisher's exact test was used as recommended (Kim 2017). The significance level was set at *p* = 0.05.

To address Research Question 3, qualitative responses from the open‐ended PEM‐CY item were analysed in their original language using MAXQDA 2024 (Version 24.10). Illustrative quotations were translated into English for reporting purposes by the first author. An inductive qualitative content analysis (Kyngäs 2020) was applied to identify recurring patterns across responses. In line with the brevity and factual nature of the responses, the analysis focused on systematic descriptive category development. Each reported strategy was treated as a meaning unit and systematically examined across responses to derive conceptually similar categories, with multiple aspects within a response assigned to separate categories where necessary. One third of the dataset was initially analysed by the first author to develop a preliminary category system through iterative comparison of text segments. The emerging categories were discussed and collaboratively refined, resulting in a preliminary codebook. The remaining data were independently assigned to the categories by both authors. If new aspects emerged, additional categories were defined, and previously coded material was revisited accordingly. Intercoder reliability indicated excellent agreement (*κ* = 0.96) (Brennan and Prediger 1981). Discrepancies were subsequently discussed, and a final version of the codebook was agreed upon (see Appendix S1). These procedures enhance the transparency and methodological rigour of the analysis.

## 3 Results

Results are presented in three sections, corresponding to the study's research questions.

### 3.1 (Pre)school Participation Patterns and Parental Desires for Change

Parents of preschool children reported slightly higher average nonparticipation rates across the five activities (19.4%) than parents of school‐aged children (11.7%) (see Table 3), though none of the individual activities showed statistically significant group differences. For both groups, the highest rates of nonparticipation were for (pre)school‐sponsored teams, clubs and organizations (47.6% for preschoolers; 30.4% for school‐aged children), followed by special roles at (pre)school (38.1% and 22.6%, respectively). Participation frequency differed significantly for two activities: Preschoolers engaged in classroom activities nearly every day (*M* = 6.49), whereas school‐aged children did so several times per week (*M* = 5.61). Conversely, school‐aged children engaged in peer interactions outside the classroom more frequently on a daily basis (*M* = 6.55) than preschoolers (*M* = 5.91). Overall, parental ratings for both groups showed relatively high involvement across all (pre)school activities, with average scores near 4 on a 5‐point scale and no significant group differences in these ratings.

**TABLE 3:** Participation patterns of preschool and school‐aged children with developmental language disorder.

Items	Never participates			Frequency (0–7)			Involvement (1–5)			Desire for change (yes)		
	% (*n*)			*M* (SD)			*M* (SD)			% (*n*)		
	Preschool (*n* = 45)^a^	School (*n* = 56)^a^	*p*	Preschool (*n* = 45)^a^	School (*n* = 56)^a^	*p*	Preschool (*n* = 44)^a^	School (*n* = 56)^a^	*p*	Preschool (*n* = 45)^a^	School (*n* = 55)^a^	*p*
Classroom activities	2.2 (1)	3.6 (2)	1.000	6.49 (1.12)	5.61 (1.73)	0.001	3.98 (1.04)	4.06 (0.90)	0.866	41.9 (18)	36.4 (20)	0.579
Field trips and (pre)school events	8.9 (4)	1.8 (1)	0.169	2.78 (1.80)	2.59 (1.79)	0.419	4.36 (0.87)	4.38 (0.76)	0.956	27.3 (12)	22.6 (12)	0.599
(Pre)school‐sponsored teams, clubs and organization	47.6 (20)	30.4 (17)	0.081	2.14 (2.44)	2.93 (2.56)	0.103	3.82 (0.85)	4.21 (0.83)	0.081	33.3 (13)	23.1 (12)	0.278
Getting together with peers outside of class	0.0 (0)	0.0 (0)		5.91 (1.51)	6.55 (1.03)	0.005	4.43 (0.79)	4.48 (0.63)	0.969	17.8 (8)	14.8 (8)	0.690
Special roles at (pre)school	38.1 (16)	22.6 (12)	0.101	3.12 (2.72)	3.81 (2.54)	0.225	4.35 (0.80)	4.22 (1.00)	0.811	22.0 (9)	22.9 (11)	0.913
Mean across activities^b^	19.4	11.7		4.09 (1.92)	4.30 (1.93)		4.19 (0.87)	4.27 (0.82)		28.5	24.0	

*Note:* *p* values are based on Fisher's exact tests, chi‐square tests or Mann–Whitney *U* tests, depending on the scale level and distribution of the variable.

^a^ The sample sizes shown in the column headers reflect the maximum number of valid responses within each dimension and comparison group. Because not all parents answered all items, the number of valid responses varies across activities within each dimension; percentages and means were calculated based on the number of valid responses per item.

^b^ Mean across activities represents the average of the item‐level values across the five participation activities.

The proportion of parents expressing a desire for change in their children's (pre)school participation was similar across preschool and school‐aged groups (see Table 3). Desired changes most often concerned classroom activities (41.9% for preschool; 36.4% for school‐aged) and (pre)school‐sponsored teams, clubs and organizations (33.3% and 23.1%, respectively). Fewest changes were noted for peer interactions outside of class (17.8% and 14.8%). Overall, parents most frequently expressed a wish for increased frequency and involvement in their children's participation in (pre)school activities (see Table 4).

**TABLE 4:** Types of desired changes in (pre)school participation.

			Type of change among parents indicating a desire for change % (*n*)									
	*n*	*n*	+frequency		−frequency		+involvement		−involvement		Different activities	
Items	Preschool	School	Preschool	School	Preschool	School	Preschool	School	Preschool	School	Preschool	School
Classroom activities	18	20	33.3 (6)	75.0 (15)	11.1 (2)	0.0 (0)	44.4 (8)	25.0 (5)	0.0 (0)	0.0 (0)	11.1 (2)	0.0 (0)
Field trips and (pre)school events	12	12	58.3 (7)	75.0 (9)	8.3 (1)	0.0 (0)	16.7 (2)	25.0 (3)	0.0 (0)	0.0 (0)	16.7 (2)	0.0 (0)
(Pre)school‐sponsored teams, clubs and organization	13	12	53.9 (7)	75.0 (9)	15.4 (2)	0.0 (0)	15.4 (2)	16.7 (2)	0.0 (0)	0.0 (0)	15.4 (2)	8.3 (1)
Getting together with peers outside of class	8	8	75.0 (6)	75.0 (6)	12.5 (1)	0.0 (0)	12.5 (1)	12.5 (1)	0.0 (0)	0.0 (0)	0.0 (0)	12.5 (1)
Special roles at (pre)school	9	11	33.3 (3)	45.5 (5)	11.1 (1)	9.1 (1)	22.2 (2)	36.4 (4)	0.0 (0)	9.1 (1)	33.3 (3)	0.0 (0)

*Note:* Only parents who indicated a desire for change were included; sample sizes therefore vary by activity.

### 3.2 Environmental Factors Affecting (Pre‐)school Participation

As shown in Table 5, parents' views on environmental influences revealed no significant group differences. Overall, 23.7% preschoolers and 33.5% of school‐aged children's parents identified clear environmental supports or barriers, with more supports (18.8% preschool; 26.1% school) than barriers (4.9% preschool; 7.4% school) reported in both groups. *Sensory qualities* (17.8% preschool; 16.1% school) and *cognitive demands* (8.9% preschool; 14.3% school) were the most frequently cited barriers. Among supports, school‐aged children's parents most frequently noted *adults' attitudes* (41.1%), whereas preschool parents cited *cognitive demands* (26.7%). *Social‐communicative demands* and *relationships with peers* were also recognized as key supportive factors in both groups. Most parents did not clearly identify environmental factors as either supports or barriers: 52.5% of preschool and 44.6% of school‐aged children's parents reported that environmental aspects were *not an issue*. Additionally, nearly a quarter of parents (23.7% preschool; 21.8% school) expressed mixed views, indicating that environmental factors sometimes helped and sometimes hindered participation.

**TABLE 5:** Perceived supports and barriers in the (pre)school environment.

	*n*	*n*	Not an issue % (*n*)		Usually helps (Supports) % (*n*)		Sometimes helps/makes harder % (*n*)		Usually makes harder (Barriers) % (*n*)		
Items	Preschool	School	Preschool	School	Preschool	School	Preschool	School	Preschool	School	*p*
Physical layout	45	56	57.8 (26)	58.9 (33)	15.6 (7)	26.8 (15)	24.4 (11)	10.7 (6)	2.2 (1)	3.6 (2)	0.209
Sensory qualities	45	56	35.6 (16)	37.5 (21)	13.3 (6)	17.9 (10)	33.3 (15)	28.6 (16)	17.8 (8)	16.1 (9)	0.902
Weather conditions	44	54	77.3 (34)	61.1 (33)	9.1 (4)	13.0 (7)	11.4 (5)	14.8 (8)	2.3 (1)	11.1 (6)	0.275
Physical demands	46	56	57.8 (26)	48.2 (27)	17.8 (8)	23.2 (13)	24.4 (11)	21.4 (12)	0.0 (0)	7.1 (4)	0.275
Cognitive demands	45	56	33.3 (15)	33.9 (19)	26.7 (12)	25.0 (14)	31.1 (14)	26.8 (15)	8.9 (4)	14.3 (8)	0.849
Social‐communicative demands	45	55	44.4 (20)	40.0 (22)	22.2 (10)	32.7 (18)	31.1 (14)	27.3 (15)	2.2 (1)	0.0 (0)	0.513
Adults' attitudes	45	56	46.7 (21)	37.5 (21)	24.4 (11)	41.1 (23)	24.4 (11)	17.9 (10)	4.4 (2)	3.6 (2)	0.370
Relationships with peers	45	55	57.8 (26)	40.0 (22)	22.2 (10)	30.9 (17)	17.8 (8)	27.3 (15)	2.2 (1)	1.8 (1)	0.309
Safety	45	56	62.2 (28)	44.6 (25)	17.8 (8)	25.0 (14)	15.6 (7)	21.4 (12)	4.4 (2)	8.9 (5)	0.383
Mean % across items			52.5	44.6	18.8	26.1	23.7	21.8	4.9	7.4	

*Note:* Not all parents answered all items; therefore, the number included in each analysis varied. The mean percentage across items represents the average item‐level percentage within each response category. *p* values are based on either Fisher's exact tests or chi‐square tests.

### 3.3 Parental Strategies to Support (Pre)school Participation of Children With Developmental Language Disorder

The inductive analysis identified 15 categories of parental support strategies in (pre)school participation. These categories reflect different emotional, organizational, academic, social and institutional forms of support. Table 6 presents the full set of categories, including frequencies and illustrative quotes. A total of 35 parents of preschool and 41 parents of school‐aged children reported one or more strategies, resulting in response rates of 77.8% and 73.2%, respectively.

**TABLE 6:** Parental strategies to support (pre)school participation.

	Preschool *n* = 35 (90 responses)		School *n* = 41 (97 responses)		
Categories of parental support strategies	% (*n*)	Rank	% (*n*)	Rank	Examples
Providing emotional and motivational support	48.6 (17)	1	48.8 (20)	1	We encourage him by saying things like, ‘You can do it!’
Communicating and preparing for activities	42.9 (15)	2	7.3 (3)	7	We explain what will happen and make sure to answer any questions she has.
Managing family time and priorities	34.3 (12)	3	9.8 (4)	6	We mark the scheduled kindergarten events in our family‐calendar.
Organizing daily tasks and logistics	20.0 (7)	4	4.9 (2)	8	We take him to kindergarten every day.
Actively engaging in (pre)school activities	17.1 (6)	5	36.6 (15)	3	I volunteer as a reading mentor at the school.
Using incentives and reward systems	14.3 (5)	6	7.3 (3)	7	Sometimes we are using rewards.
Facilitating social interaction with peers	14.3 (5)	6	7.3 (3)	7	We encourage her to maintain friendships by meeting with schoolmates outside of class.
Communicating with educators/educational institutions	14.3 (5)	6	22.0 (9)	4	We maintain regular contact with the teacher regarding his well‐being and needs.
Providing material and financial support	11.4 (4)	7	7.3 (3)	7	Contribute to the class fund for supplies, trips and celebrations.
Collaborating with other families	11.4 (4)	7	2.4 (1)	9	Asking other parents for clarification if something was not understood.
Accompanying children to (pre‐)school activities	8.6 (3)	8	12.2 (5)	5	We attend school events together.
Seeking external support and services	5.7 (2)	9	7.3 (3)	7	He attends speech therapy once a week to support his development.
Organizing access to (pre)school events	5.7 (2)	9	12.2 (5)	5	We register our child for activities.
Helping with homework and learning tasks	2.9 (1)	10	39.0 (16)	2	We practise reading with him every day.
School‐life balance	2.9 (1)	10	9.8 (4)	6	He tries out different club sessions to see which one suits him best.
Other	2.9 (1)	10	2.4 (1)	9	Vague statements; did not fit into other categories (e.g., together; raise responsibly)

*Note:* Percentages are based on the number of parents within each group; *n* indicates the number of parents reporting the category. The numbers in parentheses in the column headers indicate the total number of coded responses (multiple responses per parent possible).

*Providing emotional and motivational support* emerged as the most frequently reported category across both groups (48.6% preschool; 48.8% school). Other categories differed by group. Preschool parents most frequently reported strategies categorized as *communicating and preparing for activities* (42.9%), which were far less common in the school‐aged group (7.3%). In contrast, parents of school‐aged children more often emphasized *helping with homework and learning tasks* (39.0% school; 2.9% preschool), *active engagement in school‐related activities* (36.6% school; 17.1% preschool) and *communicating with educators* (22.0% school; 14.3% preschool). Preschool parents more frequently reported strategies categorized as *managing family time and priorities* (34.3% preschool; 9.8% school) and *organizing daily tasks and logistics* (20.0% preschool; 4.9% school). Strategies such as *seeking external support* and *school‐life balance* were rare across both groups, each reported by fewer than 10% of parents.

## 4 Discussion

Consistent with previous research on participation of children with and without disabilities (Coster et al. 2013), the highest nonparticipation rates across both groups of children with DLD were reported for school‐sponsored teams, clubs and organizations (average: 39.0%), and for special roles at (pre)school (average: 30.4%). The latter may reflect the communication demands associated with such roles, as underscored by Ekström et al. (2023), who found that students with DLD frequently experience communication‐related challenges and feelings of inadequacy or misunderstanding in school. Supporting participation in special roles may require tailored opportunities that consider children's individual communication needs and social–emotional profiles.

With regard to school‐sponsored teams, clubs and organizations, findings from organized extracurricular social activities (OESAs) are relevant as both involve structured, voluntary and socially oriented forms of participation. Although Farrell et al. (2025) found comparable participation levels between children with and without DLD in OESAs, they also reported that ability‐related factors, such as communication, motor and social skills, were associated with disengagement among children with DLD, potentially helping to explain nonparticipation in school‐based activities in the present study. In addition, the high nonparticipation in teams, clubs and organizations is likely influenced by the young age of participants. The frequent parental desire for change (average: 28.2%) and the developmental benefits of structured, voluntary activities (Larson 2000) underscore the importance of promoting access to these opportunities from an early age.

Our findings also align with those of Opitz (2024), who analysed school participation among German children with DLD, with similar levels of frequency (4.2 vs. 3.8), involvement (4.2 vs. 4.4) and parental desire for change (26.3% vs. 27.2%). These rates may appear low in comparison to Coster et al. (2013), who found that parents of elementary school children with disabilities from the United States and Canada reported a much higher desire for change across activities (69.7%) despite similarly high overall participation frequency (4.4). Consistent with the interpretation of a Swiss study, we assume that German parents of children with DLD likewise tend to value the efforts and services of the school system (Krieger et al. 2023).

Across all activities, children's involvement was relatively high, suggesting that their participation extended beyond mere attendance and aligns with a multidimensional understanding of participation (Imms et al. 2016). Descriptively, school‐aged children showed higher average scores for both frequency and involvement than preschoolers, consistent with findings in other populations (Krieger et al. 2023; Simpson et al. 2018). However, only two activities showed significant group differences in frequency, indicating broadly similar participation patterns. The higher frequency of peer interactions outside class among school‐aged children likely reflects age‐related differences and greater opportunities during nonclassroom times, though preschoolers also engaged in such interactions several times per week. Despite prior evidence of peer‐related difficulties in DLD (Lloyd‐Esenkaya et al. 2020; van den Bedem et al. 2018), parents in our study reported the highest frequency and involvement—and the lowest desire for change—in peer interactions, suggesting a potential strength and underscoring the heterogeneity among children with DLD. At the same time, differences in sample composition, including age, DLD severity and recruitment context, may explain why parents in our study reported higher peer interaction than in previous research.

Preschool children's significantly higher participation in classroom activities may reflect differences in the nature of tasks, as classroom participation in school often involves higher communicative demands (e.g., group work, discussions and presentations) than preschool routines like morning circles or storytelling. Fear of making mistakes, as reported among students with DLD (Ekström et al. 2023), may further reduce classroom engagement. Although parents of both groups reported high classroom participation frequency (several times a week to daily), this activity had the lowest involvement and highest parental desire for change. These findings highlight the crucial role of teachers in structuring classroom interactions and implementing differentiated strategies to address the diverse needs of students with DLD (Gibbons et al. 2022).

Responses to the second research question revealed that most parents did not clearly categorize environmental factors as supports or barriers, instead opting for ambiguous answers. This suggests difficulty in identifying distinct environmental influences in school contexts—a pattern also noted by Krieger et al. (2022), who attribute such responses to the context‐dependent nature of environmental factors. Nonetheless, parents reported supports over three times more often as barriers, consistent with findings in children with disabilities (Krieger et al. 2022; Coster et al. 2013). *Sensory qualities* were the most frequently mentioned barrier in both groups. Although not typically associated with DLD, emerging evidence indicates sensory challenges in this population (Simpson et al. 2020), highlighting the need for environmental accommodations (e.g., lighting and noise reduction). *Cognitive demands* were the second most reported barrier in both groups, possibly reflecting lower nonverbal cognitive abilities in children with DLD compared to typically developing peers (Gallinat and Spaulding 2014) and known difficulties with concentration and attention (Smolak et al. 2020; Yew and O'Kearney 2013). Adjustments such as extra processing time, adapting language, using visual aids and providing scaffolding may help address these challenges. At the same time, many parents also viewed *cognitive demands* as supportive, again pointing to the heterogeneity within the DLD population. Both groups identified *adults' attitudes* and *peer relationships* as major supports, underscoring the role of interpersonal relationships in promoting participation.

Findings related to the third research focus suggest that parents of preschool and school‐aged children with DLD use diverse strategies to support participation in educational settings. Framed by the fPRC (Imms et al. 2017), strategies often targeted extrinsic factors, particularly those related to environmental structuring and engagement with educational contexts. School‐aged children's parents more frequently reported engaging with the school system and communicating with educators—strategies aligned with formal school structures. In contrast, preschool parents more often focused on shaping daily routines and the logistical environment, likely reflecting the informal nature of early childhood settings as well as an increased need for support with routines of daily life in younger children. At the level of individual categories, intrinsic, child‐focused strategies emerged as the most frequently reported in both groups. Such strategies may be more immediate and accessible in everyday parenting situations than those aimed at shaping the environment or context. Preschool parents emphasized communication and (verbal) preparation that addressed the children's sense of self by helping them understand, anticipate and reflect on participation experiences. In contrast, parents of school‐aged children focused on homework and learning support, targeting activity competence. Across both groups, *emotional and motivational support* emerged as the most frequently reported category (~49.0%), consistent with previous findings (Krieger et al. 2022; Egilson et al. 2018). This reflects parental efforts to strengthen intrapersonal factors such as self‐esteem and confidence (Imms et al. 2017). Understanding how parents address both intrinsic and extrinsic factors may help professionals tailor support to align with family practices and developmental needs across educational institutions and age groups.

The findings should be interpreted in light of certain limitations. Although the sample included participants from across Germany who met defined diagnostic criteria, it was not randomly selected and did not include a control group, limiting generalizability. As participants were recruited from a rehabilitation clinic, the sample may be subject to ascertainment bias and therefore might not fully represent the broader population of children with DLD. Compared to population‐based studies reporting no significant sex differences (Norbury et al. 2016), the sample showed a skew towards boys. However, this difference was not statistically significant and may reflect clinic‐based recruitment, as boys are more likely to receive intervention services (Morgan et al. 2017). This recruitment approach may also lead to an overrepresentation of children with greater needs or better access to support; however, the considerable variability in language measures suggests that the sample captures a broad range of severity levels.

Variables potentially affecting participation in children with DLD—such as (pre)school type, socioeconomic status, severity of impairment, bilingualism and ability‐related factors (Farrell et al. 2025)—were not investigated and should be considered in future research.

As with all parent‐report measures, there is a risk for underestimation or overestimation. Although parents are well positioned to assess their children's (pre)school participation (Benjamin et al. 2017; Coster et al. 2013), some reported difficulties due to limited insight into educational settings. As parental perspectives are informed by indirect sources, they may not fully capture situational aspects of participation within classroom contexts. To address these limitations, future research should adopt a multi‐informant approach that integrates perspectives from parents, teachers and children. The complex format of the PEM‐CY also posed challenges, suggesting the need for a more accessible version. Additionally, longitudinal studies are needed to explore how participation evolves from early childhood through adolescence in children with DLD.

This study highlights the importance of understanding participation patterns and environmental influences affecting children with DLD to inform targeted, context‐sensitive interventions. Parental perspectives on participation, as captured through the PEM‐CY, contribute valuable insights into children's needs and barriers and should be considered alongside the views of teachers and children to develop a comprehensive understanding of participation in educational settings.

## Author Contributions

**Katharina Kuhlmann:** conceptualization, investigation, writing – original draft, methodology, validation, visualization, writing – review and editing, formal analysis, data curation. **Ulla Licandro:** conceptualization, writing – review and editing, validation, methodology, project administration, supervision.

## Funding

The authors have nothing to report.

## Ethics Statement

The study was reviewed and approved by the Ethics Committee of the Carl von Ossietzky Universität Oldenburg (Approval Number: Drs.EK/2022/090).

## Consent

Parental written informed consent was obtained prior to questionnaire administration and child assessments. Also, the procedure was explained in a child‐appropriate way, and verbal assent was obtained from the children before participation.

## Conflicts of Interest

The authors declare no conflicts of interest.

## Supporting information


**Table S1:** Codebook for parental strategy use.

## Data Availability

Due to privacy and ethical restrictions, the data are not publicly available but can be requested from the corresponding author.
