# Supplementary material for: Participation of Children With Developmental Language Disorders in Educational Settings—Parents' Perspectives on Patterns, Environmental Influences and Support Strategies
Source: Child Care Health Dev. 2026 Jul 3;52(4):e70310. doi: 10.1111/cch.70310 (PMC13329423; doi:10.1111/cch.70310)
Supplement: Supplementary file 1 — Table S1: Codebook for parental strategy use. [file CCH-52-e70310-s001.docx]

# **APPENDIX**

**TABLE 1.** Codebook for parental strategy use.

| **Category** | **Definition** | **Coding rule** | **Example** |
| --- | --- | --- | --- |
| Providing emotional and motivational support | Refers to actions in which parents emotionally strengthen or encourage the child, either through words or behavior, in order to build confidence, generate enthusiasm, or increase motivation. | Do not code here if the motivation is based on external rewards, if the support is practical/logistical, or if the statement is about preparing/informing the child. | - We encourage him by saying things like, "You can do it!" - Praise, encourage, build confidence. |
| Communicating and preparing for activities | Refers to parents verbally preparing, informing, or mentally guiding the child before, during, or after (pre-)school-related activities, including announcements, discussions, clarifications, or follow-up conversations. | Do not code here if the focus is emotional encouragement or learning-related help. | - We explain what will happen and make sure to answer any questions she has. - Regular conversations about school and what’s happening there. |
| Managing family time and priorities | Refers to how families plan, coordinate, and allocate their time and responsibilities to support the child’s participation in (pre-)school-related activities. | Do not code here if the statement focuses on logistical tasks, financial matters, or actual attendance at events. | - We mark the scheduled kindergarten events in our family-calendar. - Work schedules arranged so that one parent is always available for the children. |
| Organizing daily tasks and logistics | Refers to parents’ practical actions in managing everyday routines, including transportation and other daily preparations. | Do not code here if the statement refers to long-term planning or time allocation, or if logistical tasks are coordinated with other families. | - **We take him to kindergarten every day.** - Help them get dressed/ready. |
| Actively engaging in (pre-)school activities | Refers to parental involvement in (pre-)school events, projects, or routines, including volunteering, helping organize activities, participating in events, or taking on supportive roles in the educational setting. | Do not code here if the statement only refers to accompanying the child to events without active participation. | - **I volunteer as a reading mentor at the school.** - As parents, we actively participate in activities and events. |
| Using incentives and reward systems | Refers to motivating the child to participate in or complete (pre-)school-related activities by offering rewards, privileges, or special opportunities. | Do not code here if the motivation is based on emotional encouragement without tangible rewards. | - Sometimes we are using rewards. - Offer incentives for completing the task. |
| Facilitating social interaction with peers | Refers to actions by parents to enable, maintain, or strengthen the child’s relationships with peers, including arranging playdates, ensuring friends participate in activities. | Do not code here if the support focuses on emotional encouragement by family members, if the arrangement primarily involves cooperation between parents, or if the activity is leisure-focused. | - We encourage her to maintain friendships by meeting with schoolmates outside of class. - Try to involve friends in the activity as well. |
| Communicating with educators/educational institutions | Refers to parents’ communication with teachers or educators for information exchange, updates, problem-solving, or coordination regarding the child’s needs and participation. | Do not code here if the parental involvement is primarily about active participation in events. | - We maintain regular contact with the teacher regarding his well-being and needs. - Using internal school platforms to stay informed. |
| Providing material and financial support | Refers to parents supplying financial resources or material items necessary for the child’s participation in (pre-)school-related activities. | Do not code here if the provision of materials is part of daily routines or if the material or payment is linked to active parental participation. | - **Contribute to the class fund for supplies, trips, and celebrations**. - Financial support for excursions. |
| Collaborating with other families | Refers to coordination and cooperation between parents from different families to support school-related activities, such as arranging carpools, sharing information, or jointly organizing responsibilities. | Do not code here if the logistical task is organized by the family alone without cooperation with others. | - **Asking other parents for clarification if something was not understood**. - Collaboration with other parents. |
| Accompanying children to (pre-)school activities | Refers to parents being physically present with the child at (pre-)school-related activities or events without taking on active roles in organization or implementation. | Do not code here if the parents take on an active role in organizing or running the event or if the statement refers only to planning or enabling participation without physical presence. | - We attend school events together. - We accompany our child. |
| Seeking external support and services | Refers to parents arranging or using professional services or support outside the regular (pre-)school program to address the child’s developmental, educational, or personal needs. | Do not code here if the support is arranged within the (pre-)school system. | - He attends speech therapy once a week to support his development. - Socio-pedagogical family assistance. |
| Organizing access to (pre-)school events | Refers to actions taken by parents to secure their child’s participation in special (pre-)school-related activities or events, such as registering, arranging access, or ensuring eligibility. | Do not code here if the focus is on time management, practical daily preparations, or physical attendance at events. | - We register our child for activities. - It is always ensured that he can participate. |
| Helping with homework and learning tasks | Refers to parents supporting the child with schoolwork or academic practice, including explaining, reviewing, or checking assignments. | Do not code here if the focus is primarily on emotional encouragement, organizing materials or routines, or discussing activities without academic practice. | - We practice reading with him every day. - Providing support with homework. |
| School-life balance | Refers to parents enabling the child’s participation in leisure activities to maintain a healthy balance between (pre-)school demands and free time. | Do not code here if the focus is on facilitating peer contact or organizing access to a specific event for participation. | - He tries out different club sessions to see which one suits him best. - Gymnastics, taekwondo, swimming, volunteer fire department, scout. |
| Other | Used for statements that do not clearly fit into any of the established categories due to vagueness, ambiguity, or lack of specific context. | Code in this category only if the statement cannot be reasonably assigned to any other category. | - Together. - Raise responsibly. |
